# The Framework Convention on Tobacco Control in Slovakia and in Finland: one law, two different practices?

**DOI:** 10.1186/s12914-020-00243-x

**Published:** 2020-09-29

**Authors:** Barbara Pavlikova, Jitse P. van Dijk

**Affiliations:** 1 Department of Labor Law and Social Security Law, Faculty of Law, Comenius University, Šafárikovo námestie č. 6, P. O. BOX 313, 810 00 Bratislava, Slovak Republic; 2 Department of Community and Occupational Medicine, University of Groningen, University Medical Center Groningen, Groningen, The Netherlands; 3 Graduate School Kosice Institute for Society and Health, Faculty of Medicine, P.J. Safarik University in Kosice, Kosice, Slovak Republic; 4 Olomouc University Social Health Institute, Theological Faculty, Palacky University, Olomouc, Czech Republic

**Keywords:** Slovakia, Finland, FCTC, Comparison

## Abstract

**Background:**

The Framework Convention on Tobacco Control (FCTC) was ratified in 2004 in Slovakia and in 2005 in Finland. The aim of this study was to compare the implementation of the FCTC in the national laws and policies regarding smoking in Finland and Slovakia.

**Methods:**

In this case study the following areas are compared: the legal framework; the monitoring system and health promotion; treatment; and policies aimed at reducing tobacco consumption. We report on these in this order after a short historical introduction.

**Results:**

The legal frameworks are similar in Slovakia and in Finland. Finland far exceeds the minimum legal requirements. Slovakian regulations reflect the FCTC requirements; however, social tolerance is very high. In Finland the monitoring system and health promotion are aimed more at tobacco consumption. Slovakia does not follow the surveillance plans recommended by WHO so strictly; often there are no current data available. No additional documents regarding the FCTC have been adopted in Slovakia. The financial contribution to treatment is very low. Slovakian tobacco control policy is more focused on repression than on prevention, in contrast to Finland. Smoking bans meet European standards. Excise duties rise regularly in both countries.

**Conclusion:**

Implementation of the FCTC is at different levels in the compared countries. Finland has a clear plan for achieving the goal of a smoking-free country. Slovakia meets only the minimum standard required for fulfillment of its international obligations. Its policy should become more transparent by making more up-to-date data available.

## Background

Smoking is one of the most serious risk factors affecting the incidence and prevalence of major contemporary diseases with the highest morbidity and mortality [1, 2]. Projections show smoking could cause mortality of one billion victims in the twenty-first century [3, 4]. Statistics show that 9 out of 10 smokers start smoking during their teens, and if they smoke for the next 20 years, their life expectancy is 20 to 25 years shorter than that of non-smokers [5]. Before the adoption of the FCTC a growing number of smokers existed, especially in the group of teenage girls and women under 25 years. Tobacco kills more people than HIV, malaria and tuberculosis combined [6, 7]. However, based on the FCTC, the policy progress achieved between 2007 and 2010 is forecasted to result in about 7.5 million fewer smoking-related deaths by 2050 [8].

Slovakia and Finland are comparable countries with regard to the number of inhabitants and both countries have adopted the WHO Framework Convention on Tobacco Control (FCTC). Slovakia has approximately 5.4 million inhabitants [9] and Finland 5.5 million inhabitants [10]. However, in the area of tobacco control policy their results differ. Slovakia was the second country to ratify the FCTC on 4 May 2004 (no. 667/2004) [11]. Finland ratified the FCTC on 24 April 2005 [12].

The FCTC is the first international treaty negotiated under the auspices of the World Health Organization (WHO) [13]. It is one of the first global public health treaties, and also the first to tackle an individual public health threat directly [14, 15]. The Convention is legally binding for all signatories, including the European Union (EU) [16, 17].

The FCTC deals with many aspects associated with tobacco consumption. Regarding the aim of this contribution, we focus firstly on the measures aimed at reducing smoking rates such as taxation policy, smoking bans and labelling, secondly on the advertising and promotion of tobacco products, then on treatment for dependence and support for cessation, and finally on the monitoring system and surveillance.

Slovakian policies – also on smoking - are oriented towards reducing the supply of illicit drugs, so not specifically at smoking, without emphasizing harm-reduction measures, public health and well-being. The cornerstone of Slovakian policy is repression. The national strategy for the period 2009–2012 as well as the National Anti-drug Strategy 2013–2020 focused on illicit drugs and the ways they enter society [18, 19].

Prevention and early intervention play a key role in Finnish actions to prevent drug addiction, related criminal behavior and social exclusion among young people. The 1997 National Drugs Strategy sets out the principles and objectives of Finland’s drug policy, which is restricted to the illicit drugs [20, 21]. A multidisciplinary approach is emphasized. The Ministry for Social Affairs and Health (MSAH) is responsible for the national coordination of drug policy [22]. The National Drug Policy Coordination Group continued its work until 2019 [23].

Determinants of health are not systematically measured in Slovakia. All available data on key determinants, such as smoking, come from regional surveys [24]. In Finland, the National Drug Policy Coordination Group is responsible for inter-ministerial coordination in this field. It is attached to the MSAH and is composed of representatives from all relevant ministries involved in the area of drug use. The National Institute for Health and Welfare (THL) develops and directs drug prevention.

Since 2009 in Slovakia a law banning smoking in public places has been in force. It has introduced several new measures: understandable written and graphic warnings on tobacco products, their reduced availability and price rises, as well as checks on the maintenance of the ban in bars, restaurants and at public transport stops [25, 26]. In Finland a law on measures to reduce smoking was passed back in 1976. The Tobacco Act came into force in 1977 and one year later a comprehensive tobacco advertising ban entered into force and warning labels stating that smoking is hazardous to health were added to tobacco packets. Smoking in workplaces was prohibited in 1995, but bars and restaurants were omitted from the legislation. The law allowed for separate smoking premises to be set up in workplaces. The age limit for purchasing tobacco products was raised from 16 to 18 years [27]. In 2007, restaurants became smoke-free (transition period until 2009). Smoking spaces with separate ventilation could be constructed [27].

Evaluation of the success of the FCTC implementation on a detailed level is lacking in Slovakia, and the number of young smokers has a rising tendency in this country [28]. The aim of this study was to compare the implementation of the FCTC in Finland and in Slovakia by assessing the legal framework, monitoring system, health promotion and policies and measures adopted for the purpose of reducing tobacco consumption. We chose Slovakia and Finland for comparison because of their several similar characteristics. They have a very similar number of inhabitants. Both countries are Members of the EU, the Eurozone, Schengen Area and the OECD. Their state establishment is a parliamentary republic, and both have a comparable position on the Human Development Index.

## Methods

### Sample

Our design is a case study, with the history of smoking policy in Slovakia and in Finland as the subject. Documents were obtained from the official websites of national authorities (Slovak governmental institutions such as the Government Office of the Slovak Republic and the Public Health Authority and the Finnish Ministry for Social Affairs and Health and related authorities), available statistics and laws. Much useful information is published on the websites of non-governmental organizations in both countries. Statistics are available in regular WHO reports and on the official website of the EU, EUROSTAT. Laws were mainly taken from the websites www.slov-lex.sk (for Slovakia) and www.tobaccocontrollaws.org (for Finland). We also worked with secondary data, both qualitative and quantitative. For our documentary study no Ethics Committee approval was necessary.

### Measures

We measured the *legal framework* by examining the legal regulations in force in Slovakia as well as in Finland, aimed at tobacco control policy. We consulted Slovak Act no. 377/2004 on the protection of non-smokers as amended and the Act no. 106/2004 on the excise duty on tobacco products; and the Finnish Act no. 549/2016 as amended by Act no. 1374/2016 (Tobacco Act).

We measured the *monitoring system* with an overview of a number of surveys among adults and youth taken in recent years and an account of future surveillance planning.

*Health promotion* was measured according to a number of additional documents formulating health protection and prevention of tobacco use in Slovakia and in Finland. Furthermore, we analyzed existing action plans, national strategies and roadmaps.

We measured *treatment* as the type and number of available medications intended for treatment of nicotine dependency which can be purchased in a pharmacy or a general store, independently from whether they are covered by public health insurance.

*Policies and measures* aimed at reducing tobacco consumption were measured for both countries according to subcategories as follows: smoking bans, labelling, taxes and marketing. Smoking bans were evaluated from the point of view of the ban on smoking in public places. Labelling was compared according to the size of health warnings on packages. Taxes were measured as prices of lowest cost brand of cigarettes and of premium brand cigarettes in Slovakia and in Finland, as well as value added tax (VAT) and specific tax rates. Marketing was measured through a comparison of sales conditions (advertisement options and derogations) for tobacco products in Slovakia and in Finland.

### Reporting

We firstly report on the legal acts governing tobacco control policy in Slovakia and in Finland. Then we focus on the monitoring systems and tools of health promotion aimed at reducing tobacco consumption in both countries. Next we compare available treatments: the type of available medicines and the state support for such treatments. Finally we pay attention to prevention and policies aimed at reducing tobacco consumption in both countries. We focus on smoking bans in public places such as bus stops, health-care facilities, educational facilities, bars and restaurants. Labelling, tax policy in the area of tobacco products and marketing tools are also reported.

## Results

### The legal framework in Slovakia and in Finland

As an EU candidate state in the early 2000s, Slovakia had to embrace EU recommendations in many fields; regarding addiction it had a history of focusing more on public security rather than on health policies [19]. The Act no. 377/2004 on the protection of non-smokers as amended and the Act no. 106/2004 on the excise duty on tobacco products as amended are the main legal sources in the field of tobacco control. The tobacco legislation changed in 2016 and imposed new obligations on tobacco manufacturers and distributors in Slovakia. In February 2016, as a reaction to the adoption of Directive 2014/40/EU by the European Parliament and the European Council, a new amendment came into force banning the sale of 19-unit cigarette packs [29, 30]. Only packages with at least 20 cigarettes were subsequently allowed [31, 32].

The Finnish Parliament revised its existing law from 1977 into a new Tobacco Act in 2016 (Act no. 549/2016 as amended by Act no. 1374/2016). Its goal is to end the use of tobacco products and other nicotine-containing products, which are toxic to humans and cause addiction [27]. The construct ‘tobacco product’ is described as a product that can be consumed and consists wholly or partly of tobacco. The act also contains other definitions of products related to tobacco use such as different types of tobacco, emissions and marketing [33]. The objective of ending the use of tobacco products in Finland was set in 2010, as a reaction to the adoption of the FCTC in 2005. In 2012 a tobacco display ban came into force. In 2016 the 40-year-old Tobacco Act was updated as a whole. The objective of the Tobacco Act, to end the use of tobacco by the year 2030, has been extended to cover other nicotine products [27].

### The monitoring system and health promotion

Art. 20 of the FCTC deals with the question of monitoring and surveillance. The Parties should integrate tobacco surveillance systems into national, regional and global health surveillance programs so that data are comparable and can be analyzed at regional and international levels. Research, surveillance and exchange of information are critical components of the FCTC [34]. In Slovakia policies are monitored centrally, but implementation is delivered in cooperation with key stakeholders. The country has an ad hoc body coordinating drug and addiction policies: the permanent inter-ministerial Board of Ministers for Drug Addictions and Drug Control. The work of this board is the responsibility of the Anti-drug Strategy Coordination Department [18]. Slovakia does not have a special body focusing on reducing smoking and tobacco use. At governmental level in the field of tobacco control the only active bodies are the Public Health Authority of Slovakia, established in 2007 and providing general drug prevention, and the Regional Health Authorities (36), established in 2003 and providing general drug counseling including smoking and prevention of tobacco use. These organizations are subordinated under the Ministry for Health of the Slovak Republic. At non-governmental level two associations are active, both focusing on general prevention. The Slovak government’s expenditures on tobacco control in 2004 (latest available data) were 21,900 EUR (24,075 USD) [35] (4.2% GDP); however, in the budget of the Slovak Republic for 2017 we found the figure of 33,599 EUR (36,935 USD) (3.9% GDP), meant for protection and support of public health. Data specific for tobacco control are not available [36].

In Finland the Ministry for Social Affairs and Health (MSAH) is responsible for the implementation of the Tobacco Act and takes a leading role in promoting smoke-free and tobacco control initiatives. Its actions are supported by the National Supervisory Authority for Welfare and Health. The THL and the Finnish Institute for Occupational Health are the main specialist bodies involved in activities to reduce smoking [37]. The THL is also responsible, in collaboration with the Regional State Administrative Agency, for actions to reduce smoking nationwide and regionally, providing various kinds of material to authorities on the harms of smoking, and for issuing instructions aimed for example at people working with children and mass-media journalists ‘on recommendable methods for preventing and reducing smoking’ [38]. Municipalities are responsible for weaning their inhabitants off tobacco use and enforcing the sales and marketing provisions of the Tobacco Act [39]. The Action Plan on Tobacco Control (2014) stated that reports, evaluations and updates of this plan will be produced at least every five years [40]. Finland declared spending of 2,100,000 EUR on tobacco control in 2016 [35] (Table 1).

**Table 1:** Slovak and Finnish national surveys among adults and youth on tobacco use by year

Country	Adults (A) / Youth (Y)	Survey year	Tobacco type
Slovakia	A	2003	Tobacco smoking
	A	2006	Tobacco smoking
	Y	2007	Any tobacco use
	Y	2010	Tobacco smoking
	A	2010	Tobacco smoking
	Y	2011	Any tobacco use
Finland	A	2009	Tobacco smoking
	A	2010	Tobacco smoking
	Y	2010	Any tobacco use
	A	2011	Tobacco smoking
	Y	2011	Any tobacco use
	A	2012	Tobacco smoking
	A	2013	Tobacco smoking
	Y	2013	Tobacco smoking

Source: own processing based on WHO global report on trends in prevalence of tobacco smoking 2015 (2015)

In Slovakia future surveys are planned in 2020 and 2025 (adults) and in 2021 (youth). Data from planned surveys in 2015 and 2016 are not available. Surveys in Finland should take place in 2018 and 2023 (adults and youth) [41].

Health promotion in Slovakia should be supported by the National Program for Tobacco Control, the National Action Plan for Tobacco Control and by a number of state institutions and non-governmental organizations. Education should be ensured through a number of programs and campaigns with support from the state budget, and perhaps temporarily by EU funds [42, 43]. The program together with the action plan were prepared in 2007–2010 with a view to 2010, but no updated versions are available.

Finland has a separate Action Plan for Tobacco which was launched in December 2017. Together with the documents “New Era for Tobacco Control Policy: Proposals by the tobacco policy development working group of the Tobacco-free Finland 2040 network” (2013) and “Roadmap to a Tobacco-Free Finland” (2014) it has a comprehensive strategy aimed at achieving the goal of a tobacco-free country by 2030.

### Treatment and support

Promotion of smoking cessation is a very important part of building a smoking-free environment. In Art. 14 the FCTC encourages States to implement effective cessation programs, to provide accessible and affordable diagnosis and treatment of tobacco dependence and counselling services, and to establish programs in health care facilities and rehabilitation centers.

No toll-free telephone quit line or help line available for callers to discuss cessation exists in Slovakia. Diagnosis F17 (Mental and behavioral disorders due to the use of tobacco) is not on the list of priority diseases as stated in the Annex to Act no. 577/2004, and treatment costs are not reimbursed. Nicotine replacement therapies (NRTs) such as patches, chewing-gum or sprays) are sold in pharmacies without prescription. Their costs are partially covered by public health insurance when prescribed by a physician. Medicines such as Zyban, Wellbutrin and Varenicline can be legally purchased in a pharmacy only with a prescription, but they are not covered by public health insurance. Smoking cessation support is available in some offices of health professionals (psychiatrists; partially covered by public health insurance, but only by one of the four insurance companies). Neither health clinics, hospitals nor care in the community provide such support. According to the National Program for Mental Health, there was already a lack of medical staff in 2018, including psychiatrists’ surgeries.

In Finland, a toll-free telephone quit line with a person available live to discuss cessation exists. NRTs are sold in general stores without prescription. Medicines such as Zyban, Wellbutrin and Varenicline can be legally purchased in a pharmacy with a prescription. Varenicline is partially covered by national health insurance. Smoking cessation support is available in most health clinics and hospitals and in some offices of health professionals and in the community. This type of support is partially covered by national health insurance.

### Policies and measures aimed at reducing tobacco consumption

Comparing both countries, Slovakia (as one of two countries with no contact person who might provide information) dropped by three places between 2013 and 2016, namely from 27th to 30th place with a total score of 41 points. Finland improved by three places, from 9th to 6th place with a total score of 60 points [44]. The guideline principles contained Art. 4 of the FCTC have to be reflected in all policies related to tobacco control and public health.

Slovakian tobacco policies rank low compared to the other EU countries [45]. Despite not involving decentralized structures and other stakeholders in the decision-making process, selective and indicated prevention and treatment policies in Slovakia are implemented and delivered by non-profit organizations and private centers [18]. Measures taken to ensure national tobacco control can be divided into four groups in general: legislative measures, tax measures, education, and counseling and medical care [46]. Legislative measures are mentioned in the part of this paper dealing with health promotion. Counselling and medical care are entrusted to regional offices of public health and to physicians in the field of psychiatry [42, 43].

Finnish tobacco policies rank high compared to the other EU countries [45]. Foreign tobacco company sales have been in decline since 2012 in Finland, and they are being attacked from several directions. Firstly, education on the harm caused by cigarettes has led Finns to slowly drop the habit of smoking, as health awareness has become stronger. Secondly, strict legislation and increasing prices have lowered consumption, although private imports of tobacco products from cheaper countries have increased [47].

### The smoking ban

Article 8 of the FCTC deals with smoking bans. States should provide protection from exposure to tobacco smoke in indoor workplaces, public transport, indoor public places and possibly other public places.

According to the Finnish Tobacco Act 2018, smoking is prohibited in indoor areas of buildings, vehicles or similar places which are accessible to the public or employees or accessible to customers for the purpose of providing commercial or public services. It is also prohibited in indoor and outdoor areas of day-care centers or facilities providing pre-school, elementary or secondary school education, as well as in all restaurants and in shared and common indoor areas of housing corporations [48–51]. However, this ban is not always complete.

The situation in Slovakia is very similar. This means that for example in restaurants, bars or governmental facilities a special room / space for smokers can be designated. In Slovakia, according to the Act on Protection of Non-smokers, smoking is not permitted in health-care and educational facilities, in indoor offices and workplaces (governed by the Act on Occupational Safety and Health Protection 2006) and only partially in restaurants and governmental facilities, where a smoking room or smoking space can be created. Any smoker must be at a distance of at least four meters from public transport stops [52] (Table 2).

**Table 2:** Smoke-free environments in 2017 in Slovakia and in Finland

Complete^a^ smoking ban exists in:	Slovakia	Finland
Health-care facilities	Yes	No
Educational facilities except universities	Yes	Yes
Universities	Yes	No
Government facilities	No	No
Indoor offices and workplaces	Yes	No
Restaurants	No	No
Cafés, pubs and bars	No	No
Public transport	No	No
All other public places	–	–

^a^Complete means that smoking is not permitted, with no exceptions allowed, except in residences and indoor places which serve as equivalents to long-term residential facilities (e.g. prisons and nursing homes). Ventilation and designated smoking rooms / areas do not protect people from the harms of passive smoking, and the only laws providing protection are those that result in the complete absence of smoking in all public places

Source: own processing based on WHO report on the global tobacco epidemic 2017 (2017)

### Labelling

The FCTC also responds to the question of labelling of packages of tobacco products. It requires concrete steps by stipulating the form of health warnings and banning false impressions of harmful effects, to ensure the provision of true information on the effects of tobacco.

In May 2016 the Slovak Government adopted the EU legislation on tobacco packaging; cigarette-pack labelling rules were changed, introducing larger health warnings as well as several other significant modifications [29]. Tobacco products, electronic cigarettes, refill containers and herbal products for smoking as well as nicotine liquids and nicotine-free liquids intended for “vaping” can only be sold in retail packaging meeting the requirements set in legislation [27, 33, 53]. There is a graphic type of warning with 65% of the pack covered. Finland applied the same regulations [54, 55].

### Taxes

Taxation policy as an effective means of reducing tobacco consumption is set out in Art. 6 of the FCTC. Parties to the Convention should implement tax and pricing policies, if appropriate, to support health objectives.

In 2016 the Finnish Government received 975 million EUR as tobacco tax revenues from the total tax revenues of 52,012 million EUR. Tobacco tax is collected on cigarettes, cigars, cigarillos and roll-your-own tobacco as well as other products containing tobacco and rolling paper. Since the beginning of 2017, tobacco tax has also been collected on nicotine and nicotine-free liquids for electronic cigarettes. Tobacco tax was raised at the beginning of January 2018 and the next increase came at the beginning of July 2018 [56]. This tax was previously also raised at the start of the years 2009, 2010, 2012 and 2014. Since these years smoking has decreased, revenues from tobacco excise duty have increased and there has been no increase in private imports of cigarettes [40]. In 2017 a working group was appointed by the MSAH; it should contribute to the formulation of tobacco policy in such a way that the use of tobacco should end in Finland by the year 2030 [57].

In 2016, the Slovak Government received 673 million EUR in tobacco tax revenues from the total revenues of 11,070 million EUR [58]. Tobacco products on which tax is levied are cigarettes, cigars and loose tobacco. Other tobacco products include those not consumed during any burning process, except chewing tobacco and snuff, as well as tobacco raw material [59]. Increases in taxes are irregular [60]. A further increase was planned for February 2019 [58]. As of 1 January 2018, the overall minimum excise duty on tobacco products (excluding VAT) was 62.54% of the weighted average price in Slovakia and 69.57% in Finland [61].

### Marketing

Advertising and promotion of tobacco products is governed by Art. 13 of the FCTC. It suggests a comprehensive ban on all tobacco advertising, promotion and sponsorship, and lays down the minimum required standards.

Growth in sales of tobacco products in Slovakia used to be significantly supported by marketing activities and advertising ranging from materials in printed press form to posters, banners and specialised stands in shopping malls, for example. Attracting smokers’ attention by offering various prizes through competitions in which smokers can participate was the main highlight in 2016. Increasing brand awareness and product innovation was also increasingly popular due to mobile stands and well-trained staff. Internet retailing of products containing tobacco is not permitted in Slovakia [29]. Act no. 147/2001 on Advertising banned tobacco product advertisements in all forms of information media. There are derogations concerning the designation of specialized stores, use of trademarks, issuing of leaflets, brochures and other publications intended exclusively for producers and traders and consumer information [62].

In Finland, the Tobacco Act 2018 bans all advertising of tobacco products (including e-cigarettes). Derogations concern marketing in publications which are printed and published outside the EU and are not principally intended for the EU market, and some other exceptions. It is prohibited to display tobacco products in retail sales premises [27]. The country has also considered placing an 18-year age restriction on films and video recordings presenting tobacco products, imitations, smoking scenes and smoking. This provision should not apply to films clearly presenting the health risks of smoking, or films showing historical figures smoking in a natural context [63] (Table 3).

**Table 3:** Data on labelling, taxes and marketing by country

	Slovakia	Finland
Labelling		
Different?	According to EU legislation	According to EU legislation
Taxes		
Total	€ 11,070 × 10^6^ (12.3% GDP)	€ 52,012 × 10^6^ (24.1% GDP)
Smoking	€ 673 × 10^6^ (0.75% GDP)	€ 975 × 10^6^ (0.45% GDP)
Marketing		
Banned?	Not banned	Banned

Source: own processing (2019)

## Discussion

We have explored the differences in the implementation of the FCTC in Slovakia compared to Finland. Regarding the legal framework, we found that Slovakian policy and legislation fulfills the minimum of European and global requirements, while Finnish policy largely exceeds those requirements. Regarding monitoring systems and health promotion, we found that Slovakia lacks a comprehensive and comprehensible strategy in the field of tobacco control, as well as specialist institutions. Treatment is available in both countries, but health insurance coverage in Slovakia is absent or lower. Medical and community support is not as developed in Slovakia as it is in Finland. Slovakian policy is focused on repression, and Finnish policy on ending tobacco consumption. The definition of a tobacco product is very similar in both countries, and special legislation on tobacco products has been adopted. Both countries have introduced smoking bans and customized their tax policies. The EU could play a very important role especially in the field of stricter control of fulfilment of the Member States´ obligations, because if a State is not willing to do more than the minimum, some kind of motivation can be supportive. If the motivation does not work, sanctions can also be considered.

We found that legislation aimed at reducing smoking does effectively exist in both countries. In Slovakia however, in contrast to Finland, the legislation is poorly enforced. There is noticeable inconsistency in enforcement particularly in the areas of sales of cigarettes to juveniles and smoking in public places such as public transport stops. Smoking in Slovakia is very widely tolerated by society, and parents often smoke together with their children or in their presence from birth. However, this is difficult to prove objectively; there are no complete studies and this statement is based more on direct and long-term observation on the streets [64]. Studies tend to classify family smoking for example as one of the factors inducing young people to smoke [65, 66]; in Finland this is not accepted to such an extent. Paveleková and Peterková [67] state that people’s own smoking significantly affects their attitude towards smoking by others. The higher the number of active smokers in the country, the more tolerant the attitude of society to smoking by others. Active smokers are also more reluctant to respect stricter regulations and smoking bans [68, 69]. These features explain why the tolerance towards smoking is higher in Slovakia than in Finland, where the numbers of active smokers are much lower. Effective control mechanisms and treatment support can lead to decreasing numbers of smokers. Examples of other countries such as Sweden, Norway and Slovenia show that a lower number of smokers, based on effective tobacco control policy, increases the enforceability of the legal measures and decreases the public tolerance towards smoking [70–74]. Such legal measures are easily introduced; however they should be properly implemented and enforced as well.

Our findings show the absence and non-functioning of governmental and non-governmental bodies in Slovakia which should jointly implement programs to prevent smoking. Of all the governmental authorities mentioned in the FCTC there is not one which really functions. Only the Public Health Authority of the Slovak Republic and the Regional Health Authorities are active, but they have more responsibilities and are not aimed at tobacco control alone. In Finland special governmental and non-governmental bodies were created to perform their roles in the process of achieving smoking-free status. As experience from other countries such as the Pacific islands and Brazil shows, strong support by the authorities at governmental and non-governmental levels and especially developing infrastructure capacity contributes to achieving successful results in tobacco control policy [75, 76]. Protection from interference by the tobacco industry also represents a very important part of tobacco control policy [77, 78]. Generally speaking, based on the presence of governmental and non-governmental bodies in line with the above examples, one might expect some decrease in the number of smokers [79], and consequently also in societal tolerance.

We further found with regard to monitoring systems and health promotion that no special institution dealing with tobacco control policy exists in Slovakia. Only a general anti-drug policy is present. Documents on tobacco control have not been further developed since 2010. Finland has a complex structure of institutions aimed at achieving a smoking-free environment. The presence of a special political strategy consisting of a package of documents such as a national plan, action plan and a roadmap confirms this goal. Singapore has also made this move [80], and achieved far better health outcomes than other countries.

Finland only needs to evaluate and develop what already exists in order to produce successful results, so the political burden is much lower than in Slovakia. In central Europe, the history of smoking is longer than the anti-tobacco measures, and societal tolerance is much higher than in Scandinavia [81–83]. Slovakia needs to return to the beginnings of the FCTC and to bring into operation some functional institutions providing regular monitoring, and create a transparent system of documents considering the current situation of the smoking environment.

Regarding treatment and support we found that in Slovakia no telephone quit line exists, unlike in Finland. No special support is provided in hospitals or in the community. NRTs can be purchased with or without prescription in pharmacies. With a prescription they are partially covered by public health insurance. Medicines against smoking, also on prescription, are not covered. In Finland, NRTs are sold in stores and medicines in pharmacies, and with a prescription they are partially covered by national health insurance. There is also official support for cessation provided in hospitals and in the community. Countries with the highest level of achievement in the field of tobacco dependence treatment, such as Australia, Canada or Denmark [84], strongly support the public availability of medical treatment and NRTs, together with the possibility of health insurance coverage. There also exist national quit lines [79, 85–87]. In Slovakia, it is necessary to strengthen the supportive network of medical staff and community provision to increase the impact of anti-tobacco policy. Financial coverage from public health insurance – full or partial – also motivates smokers in the cessation process.

Finally, in the field of policies aimed at reducing tobacco consumption, we found that smoking bans are stricter on paper in Slovakia, and that cigarette pack labelling does not differ from that found in Finland. Tobacco taxes are paid on the same products in both countries, but Finland has higher revenues from tobacco tax. In Finland taxes are increased on a regular basis, but only irregularly in Slovakia. Overall minimum excise duty is higher in Finland. With regard to advertising bans on tobacco products the differences are not significant. Exceptions from bans are more specific and cover more areas in Finland. Increasing tax and banning tobacco product advertising have been implemented successfully in both countries, but the number of daily smokers is not falling commensurately in Slovakia. However, according to the World Bank, increasing the price of tobacco through taxation is an effective way of reducing smoking in the population [88]. The high price discourages young people from taking up smoking, reduces the number of cigarettes smoked, increases the number of those who quit smoking as well as attempts to quit, and reduces health differences between demographic groups [89–91]. Bans on tobacco advertising can also lead to reduction in its consumption [92–94]. Other findings show that a comprehensive set of tobacco advertising bans is more effective than only a limited set of restrictions [95, 96].

### Strengths and limitations

Slovakia and Finland have several similar characteristics which allow us to compare their tobacco control policy. Both countries are EU Member States and included in the Eurozone, Schengen Area and OECD. They are both parliamentary republics and as we have already mentioned, they have a comparable number of inhabitants as well as Human Development Index [97, 98].

We have compared the implementation of the FCTC in Slovakia and in Finland and described the recent situation, which had previously been unclear. Finland is one of the best European examples of successful policy development in the tobacco control field and can be used as an example of good practice. We have presented an overview of the issue of smoking reduction policy in Slovakia. This is intended to help raise the possibility of further comparison with other countries and to deepen the process of better implementation of the FCTC in Slovakia.

We consider the lack of current data, recent statistics and comparable studies in Slovakia to be the biggest limitation. The country has not enough or no up-to-date sources, publications or figures which could contribute to a better understanding of the current status of FCTC implementation. The very few available sources are often outdated and published solely in Slovak language. Their formulation is very vague and often only copies the general political declarations. Specific strategies and plans for further steps are lacking. The results of recent surveys, if there have been any, are not presented publicly. The information provided by the Slovakian authorities for international reports are only partial and do not allow us to create a real picture of the situation in the country.

### Implications

Our findings in the field of the FCTC implementation process in Slovakia, in comparison with Finland, help us to get a better idea of the current situation in terms of policy aimed at reducing tobacco consumption. They point out the most problematic areas and shortcomings in the implementation procedure. Their presentation not only in Slovak language will contribute to the possibility of broader international cooperation. Policy in Slovakia needs to be changed in a way that allows the establishing of a functional complex of governmental and non-governmental institutions supported with a financial framework, of the kind that will raise public awareness of the negative consequences of smoking. Those institutions should focus on implementing the approved policy measures. The adoption of a specific national anti-tobacco program, supported by well-apportioned funding and management should contribute to the achievement of improvement in the overall health status of the population. Regular national surveys would help to adjust the legal framework and enable it to react to societal needs. Our findings show that high prices, taxation, labelling and advertising bans themselves are not sufficient for reducing the smoking prevalence rate. They represent a good start on the way to greater achievement. However, these measures should be supported by a functioning monitoring system, consistent and ongoing financing and accompanied by a sufficient number of well-trained medical staff. The international experience is proof that sustainable results stem from a combination of well-set institutions and financial sources, and from the perception of smoking and its health impacts in the wider context of public health. Furthermore, the EU (a signatory of the FCTC as well) could take better care of what its Member States are doing to implement the FCTC.

Further research has to be done for the purpose of identification and naming of the main barriers in political, financial and other areas, which do not allow more extensive development in real implementation of the FCTC. Next, better insight into compliance is needed. It is difficult to induce citizens to respect health norms if the state itself ignores them and creates only virtual boundaries which cannot be effectively monitored. It is also necessary to identify the most effective tools, which have helped Finland to achieve the present progress in reducing smoking among all age groups.

## Conclusion

Finland is the first country in the world to set up a tobacco-free society as a legislative objective. Finland was thus the first country to move from an official policy of reducing the consumption of tobacco products to a policy aimed at ending their use altogether [40]. Finland has done much more than Slovakia in the tobacco control area, although Slovakia has made progress on tobacco control in recent years. However, Slovakia can still do more to make the proven tobacco control tools work for its citizens’ well-being [54]. Institutional support of smoking prevention and tobacco control is at a very low level in Slovakia. Measures aimed at fighting smoking include several steps: raising awareness among the population, limiting the sale of tobacco products, and banning smoking in public places [99].

Member states of WHO have adopted a voluntary global target to reduce tobacco use (smoking and smokeless) by 30% by 2025. According to WHO, based on current smoking trends, Slovakia will not achieve the smoking component of the target, but Finland will [39]. According to Puska [100] the best implemented articles of the FCTC are those concerning smoke-free areas, labelling of tobacco products and sales to minors. Countries with available data show that there was an overall decline in smoking prevalence between 2005 and 2015, and that countries with stronger implementation of the FCTC articles had significantly greater decline in smoking [101].

## Data Availability

The corresponding author can provide material used upon reasonable request.

## Abbreviations

EU: European Union
FCTC: Framework Convention on Tobacco Control
MSAH: Ministry for Social Affairs and Health (Finland)
NRTs: Nicotine replacement therapies
THL: National Institute for Health and Welfare (Finland)
VAT: Value Added Tax
WHO: World Health Organization
